# Curve severity and apical vertebral rotation and their association with curve flexibility in adolescent idiopathic scoliosis

**DOI:** 10.1007/s12306-020-00660-0

**Published:** 2020-04-22

**Authors:** S. P. Mohanty, M. Pai Kanhangad, A. Gullia

**Affiliations:** Division of Spine Surgery, Department of Orthopaedics, Kasturba Medical College, Manipal Academy of Higher Education, Manipal, Karnataka 576104 India

**Keywords:** Adolescent idiopathic scoliosis, Apical vertebral rotation, Nash–Moe index, Cobb’s angle

## Abstract

**Purpose:**

To determine the association between coronal Cobb’s angle and Nash–Moe index in patients with adolescent idiopathic scoliosis. We also attempted to determine whether apical vertebral derotation depended upon the curve flexibility.

**Overview of literature:**

The three-dimensional nature of adolescent idiopathic scoliosis (AIS) is well established. Knowledge of all components of this complex deformity is essential to formulate effective treatment strategies. Though the importance of quantifying all the components of the deformity, in AIS, has been analysed in detail, very few studies have been done to ascertain the relationship between the coronal plane deformity and apical vertebral rotation.

**Methods:**

Digitalised standing and supine stretch anteroposterior (AP) radiographs of 158 patients with AIS were analysed. The standing and supine stretch AP radiographs were compared to calculate the percentage reduction of Cobb’s angle to determine curve flexibility. The derotation of the apical vertebra on application of traction was also noted. The one-way repeated ANOVA was used to determine the association between Cobb’s angle and Nash–Moe index. The independent sample *t* test was used to determine whether a statistically significant difference was present, in the age of the patient, severity of the curve and percentage reduction of Cobb’s angle between those curves that derotated and those that did not, when stretched.

**Results:**

The one-way repeated ANOVA revealed an association between Cobb’s angle and Nash–Moe index on the standing and supine AP stretch radiographs (*P* < 0.01). The Independent sample *t*-test showed a statistically significant difference in percentage reduction of Cobb’s angle between those curves that derotated compared to those that did not, on stretch (*P* < 0.01).

**Conclusions:**

This study demonstrates that there is an association between apical vertebral rotation and the coronal plane deformity. It also demonstrates that flexible curves derotate to a greater extent compared to rigid curves, when stretched.

## Introduction

Idiopathic scoliosis is the commonest type and accounts for nearly 80% of all structural scoliosis [1]. The three-dimensional nature of adolescent idiopathic scoliosis (AIS) is well established. Apart from the obvious lateral curvature, the spine is hypokyphotic at the apex and the vertebral bodies rotate towards the convexity of the curve [2]. Knowledge of all components of this complex deformity is essential to formulate effective treatment strategies. Early surgical principles primarily addressed the correction of the coronal plane deformity by distraction [3]. In recent times, there has been a growing consensus among surgeons, to address the three-dimensional nature of this deformity [4, 5].

The severity of the curve is routinely measured on an anteroposterior (AP) radiograph. The Cobb’s angle is considered to be the gold standard in measuring the lateral curvature, as it closely corresponds to the magnitude of the deformity [6]. It is easy to perform, with a low intra- and interobserver variability [7]. Measurement of apical vertebral rotation (AVR) is an integral part of AIS evaluation, as it predicts risk of progression and is necessary for planning levels of instrumentation. Various methods have been described to determine AVR, most of which are based on the evaluation of the relative position of the posterior elements [8, 9]. Cobb’s method is not reliable as it does not correlate with vertebral rotation in degrees. The Perdriolle method is less reliable when the vertebral rotation is greater than 30° [10]. While the computed tomography-based methods allow a more precise measurement of vertebral rotation, it requires a prohibitively high radiation dose [11]. Using predefined vertebral shape parameters like the angle from the pedicle to the spinous process and the distance from the pedicle to the vertebral body centre, Drerup developed a trigonometrical model to measure AVR [12, 13]. Even though the Nash–Moe index is an approximate measure of vertebral rotation, it is still one of the most popular methods used in clinical practice [14]. Recognition of the sagittal plane deformity has improved the understanding of AIS and has been integrated into newer classification systems [15].

Though the importance of quantifying all the components of the deformity, in AIS, has been analysed in detail, very few studies have been done to ascertain the relationship between the coronal plane deformity and AVR. This study was carried out to determine the association between coronal Cobb’s angle and Nash–Moe index in patients with AIS. We also attempted to determine whether the reduction in AVR depended upon the flexibility of the curve.

## Materials and methods

In a retrospective study, 354 consecutive patients presenting with scoliosis between the age of ten and eighteen years, from January 2012 to December 2017, were analysed. A clinical examination was done to detect neurological deficit, congenital anomalies and other causes of scoliosis. Standardised standing AP and lateral radiographs, supine lateral bending and stretch AP radiographs of the whole spine were obtained in all patients. They also underwent magnetic resonance imaging of the whole spine to rule out craniovertebral and spinal cord anomalies. One hundred and seventy-eight cases of congenital, neuromuscular, scoliosis secondary to tumours and infection were excluded. Twenty-seven patients with craniovertebral and cord anomalies were also excluded. Thus, 158 patients with AIS formed the basis of this study. Institutional ethical committee clearance was obtained prior to the commencement of the study.

### Radiological evaluation

Digitalised radiographs were analysed by two independent observers (MPK and AG) in a blinded fashion using RadiANT software version 4.6.5. In case of a difference in the two readings, the decision of the senior observer was considered to be final. The upper end, lower end and apical vertebrae were identified on the standing AP radiographs. All curves were classified according to the Lenke’s classification [15]. The severity of the curve was measured using the method described by Cobb et al. [6]. The Nash–Moe index was used to determine the rotation of the apical vertebra [8]. Similar method was used to determine the severity of the curve and AVR in the supine stretch film. The standing and supine stretch AP radiographs were compared to calculate the percentage reduction of Cobb’s angle to determine curve flexibility. The derotation of the apical vertebra on application of traction was also recorded. In cases with more than one curve, these measurements were taken for all the structural curves. Thus, 196 curves in 158 patients were analysed. The Risser index was noted in all patients [16].

### Statistical analysis

All statistical analyses were done using Statistical Package for Social Sciences for Windows version 18. The intraclass correlation and kappa coefficients were used to determine the intra- and interobserver variability for Cobb’s angle and Nash–Moe index, respectively. Continuous variables like age, Cobb’s angle and percentage reduction of Cobb’s angle were expressed as mean ± SD. Percentages were used to express categorical variables like Nash–Moe index. The one-way repeated ANOVA was used to determine the association between Cobb’s angle and Nash–Moe index on standing and supine stretch AP radiographs. It was also used to compare the Cobb’s angle in the thoracic, thoracolumbar and lumbar spine. Post hoc Tukey’s test was done to determine whether a statistically significant difference was present between the various Nash–Moe grades. Spearman correlation coefficient was used to determine the relationship between age of the patient, severity of the curve and percentage reduction of Cobb’s angle on stretch. The independent sample *t*-test was used to determine whether a statistically significant difference was present, in the age of the patient, severity of the curve and percentage reduction of Cobb’s angle between those curves that derotated and those that did not, on stretch. The Chi squared test was used to compare the frequency of the various Nash–Moe grades in the thoracic, thoracolumbar and lumbar spine. It was also used to compare the frequency of Risser 0, 1, 2 incurves that derotated and those that did not, when stretched. Receiver operating characteristic (ROC) of the curve was performed to assess the area under the curve (AUC) of age, standing Cobb’s angle and percentage reduction of Cobb’s angle in relation to apical vertebral derotation. A *P* value less than 0.05 was considered to be statistically significant.

## Results

The inter- and intraobserver agreeability for Cobb’s angle was 0.93 and 0.98, and for Nash–Moe index, it was 0.61 and 0.86, respectively. The mean age of patients was 14.5 ± 2.1 years. The male-to-female ratio was 1:6.75. The most common curve pattern was Lenke type 1B. The ratio of right-sided to left-sided curves was 2.37:1. The most common apical vertebrae were T9, L1 and L2 in the thoracic, thoracolumbar and lumbar spines, respectively. The mean Cobb’s angle was 51.26 ± 17.43° and 28.28 ± 14.97° on the standing and supine stretch AP radiographs, respectively. Supine stretch AP radiographs showed a 46.2 ± 16.1 percent reduction of Cobb’s angle. The details of curve severity, flexibility and AVR are shown in Table 1.

**Table 1:** Curve severity, flexibility and apical vertebral rotation

	Thoracic (*n* = 113)	Thoracolumbar (*n* = 38)	Lumbar (*n* = 45)	*P* value
Cobb’s angle (mean ± SD)	55.41 ± 17.16	45.87 ± 17.27	45.39 ± 15.56	< 0.01*
Traction Cobb’s angle (mean ± SD)	32.5 ± 15.65	20.94 ± 10.39	23.85 ± 12.91	< 0.01*
Percentage reduction of Cobb’s angle	42.42 ± 15.69	54.28 ± 15.18	48.92 ± 15.11	< 0.01*
Standing radiographs				
Nash–Moe grade 1	42	8	12	0.477**
Nash–Moe grade 2	62	23	30	
Nash–Moe grade 3	9	7	3	
Supine stretch radiographs				
Nash–Moe grade 1	78	22	27	0.119*
Nash–Moe grade 2	30	15	17	
Nash–Moe grade 3	5	1	1	

*One-way repeated ANOVA, **Chi squared test

The one-way repeated ANOVA revealed an association between Cobb’s angle and Nash–Moe index on the standing and supine stretch AP radiographs (*P* < 0.01). Post hoc Tukey’s test showed a statistically significant difference in Cobb’s angle between the three Nash–Moe grades, on the standing as well as supine stretch AP radiographs (*P* < 0.01). A higher Nash–Moe index was associated with a higher Cobb’s angle. Bivariate analysis revealed a weak negative correlation between Cobb’s angle on standing AP and percentage reduction of Cobb’s angle on supine stretch AP radiographs (Spearman’s rho = − 0.243, *P* = 0.01); however, there was a poor correlation between age and percentage reduction of Cobb’s angle (Spearman’s rho = − 0.131, *P* = 0.07). In the supine stretch AP radiographs, the reduction in Cobb’s angle was accompanied with a reduction in Nash–Moe index in a total of 77 curves, whereas in 119 curves it showed no change. The Independent sample *t*-test showed a statistically significant difference in percentage reduction of Cobb’s angle between those curves that derotated compared to those that did not, when stretched (*P* < 0.01). However, no statistically significant difference in age and Cobb’s angle was noted between the two groups [Table 2]. The frequency of Risser 0, 1, 2 was higher in curves that derotated compared to curves that did not (46.75% vs 27.73%) (*P* = 0.018).

**Table 2:** Factors affecting curve derotation

	Curves that derotated by one Nash–Moe grade (*n* = 77)	Curves that did not derotate (*n* = 119)	*P* value
Age	14.23 ± 1.68	14.68 ± 2.28	0.142*
Cobb’s angle (standing AP)	51.98 ± 17.64	50.8 ± 17.36	0.644*
Percentage reduction of Cobb’s angle	50.66 ± 13.69	43.34 ± 16.93	0.002*

*Independent sample *t*-test

**Fig. 1:**
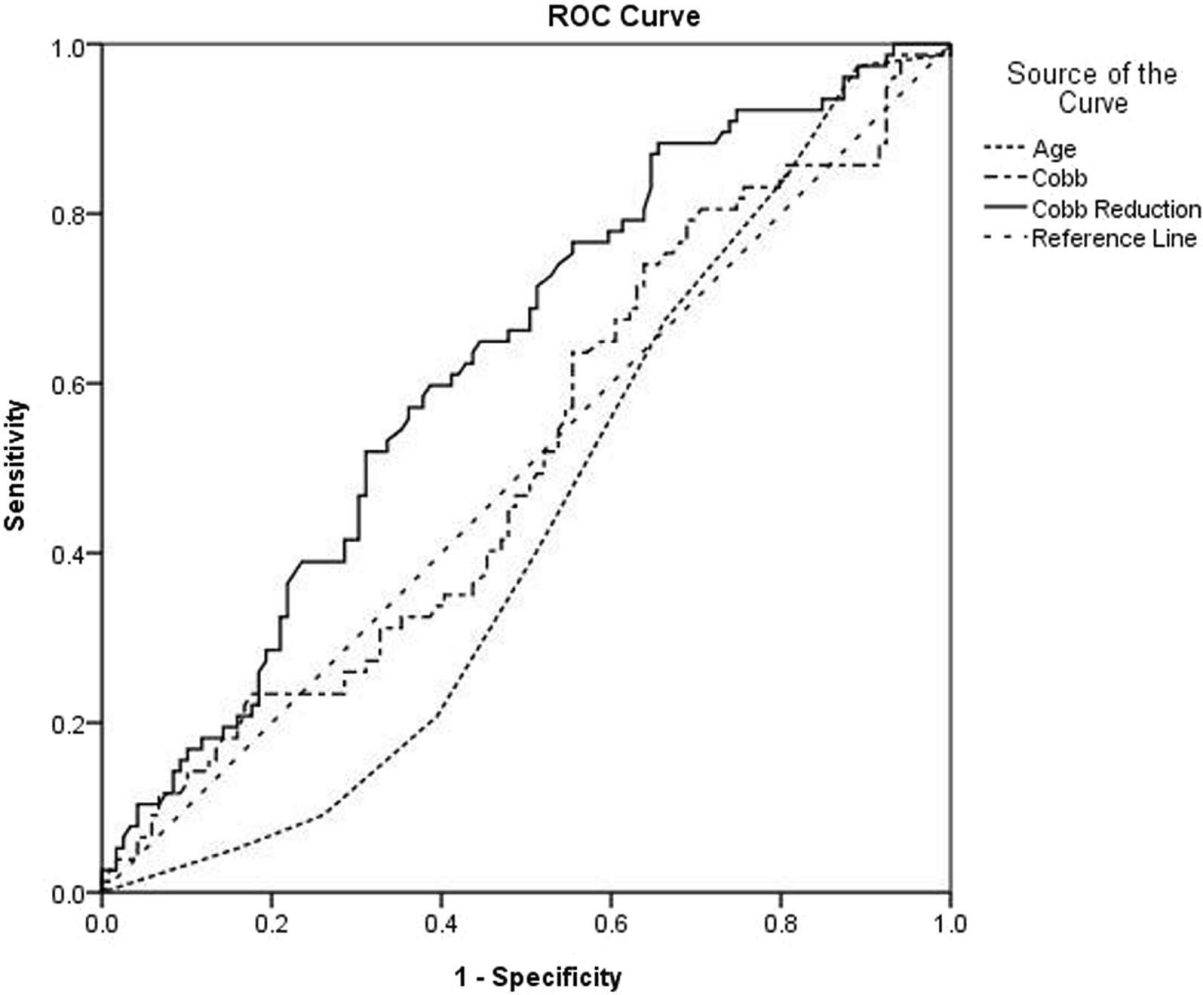
Receiver operating characteristic curves for age, standing Cobb’s angle and the percentage reduction of Cobb’s angle in relation to apical vertebral derotation

The ROC analysis revealed AUC of 0.44, 0.51 and 0.63 for age, standing Cobb’s angle and the percentage reduction of Cobb’s angle in relation to apical vertebral derotation [Fig. 1]. The critical value of 48 percentage reduction of Cobb’s angle had a sensitivity of 61%, a specificity of 59%, a positive predictive value of 70% and a negative predictive value of 49%, for derotation of the apical vertebra by one Nash–Moe grade, when stretched.

## Discussion

The three-dimensional nature of scoliosis was well understood prior to the invention of radiography [2]. With the advent of radiographic evaluation of scoliosis, greater emphasis was assigned to the coronal plane deformity. In 1966, Scoliosis research society adopted the use of Cobb’s angle for quantitative assessment of severity of the curve due to its reliability. The current study showed high inter- and intraobserver agreement for Cobb’s angle, similar to that of previous studies [7, 11]. This study showed significantly less severe curves in the thoracolumbar and lumbar spines. This is similar to the findings of other authors [17]. While various techniques have been described to quantify the rotational deformity, the Nash–Moe index is still widely used in clinical practice. This study demonstrated that there was no significant difference in the frequencies of various Nash–Moe grades in the thoracic, thoracolumbar and lumbar spines. This is consistent with the findings of Stoke et al.[18].

The origin and progression of the deformity in AIS are not well understood. Various theories have been proposed regarding the development and progression of the rotational deformity in scoliosis. Lovett and Arkin observed that coupling of lateral bending and axial rotation is a kinematic property of the normal spine [19, 20]. While studies have shown that there is some nonlinear relationship between the elemental components of the deformity, its exact nature is not clear. Our study showed that there was a statistically significant difference in the Cobb’s angle between the various Nash–Moe grades, and the higher Nash–Moe grades had higher Cobb angles. This is similar to the findings of Sullivan et al. and Morrison et al., who found a correlation between Cobb’s angle and AVR [21, 22].

This study demonstrated that more severe curves were less flexible, while age did not significantly affect curve flexibility. Mathematical models have shown that ribs increase the stiffness of the thoracic spine in scoliosis [23]. This probably explains why the reduction of the coronal Cobb, upon stretch, was significantly lower in the thoracic compared to the thoracolumbar and lumbar spines, in this study. Kadoury et al. studied the effects of various types of instrumentation on vertebral rotation. They observed that though some derotation was seen with Harrington distraction instrumentation, better correction was possible with newer techniques [17]. In the current study, 77 curves derotated by one Nash–Moe grade, whereas 119 curves showed no change upon stretching. Curves that derotated had a significantly higher flexibility compared to curves that did not. However, no significant difference in age and Cobb’s angle was noted between the two groups. ROC analysis showed that curves with coronal Cobb that reduced by 48% in supine stretch films had a tendency to derotate by one Nash–Moe grade with a sensitivity of 61% and a specificity of 59%.

Early surgical principles primarily addressed the lateral curvature of the spine. Though satisfactory results were obtained with Harrington’s distraction instrumentation, the current study showed distraction alone did not completely address all components of the deformity [17]. Cotrel and Dubousset shifted the premise of curve correction from distraction to derotation of the curve about the axial plane [4]. However, various studies have shown that this concept is not entirely correct. Thus, newer correction manoeuvres that employ a combination of derotation and translational forces may achieve better three-dimensional deformity correction and restoration of global spinal alignment [24]. Timing of surgery is also important in achieving optimum correction of the deformity [25]. Therefore, surgical correction should be carried out before the curve progresses in severity and becomes rigid.

The strengths of this study include its large sample size and detailed statistical analyses, some of which have not been previously done. While the Nash–Moe index is commonly used in clinical practice, it is only a rough estimate of AVR with a 25° interval between each grade. Being a complex three-dimensional deformity accurate measurement of vertebral rotation is difficult. Another drawback of the current study is the inability to standardise the stretch force applied while obtaining supine AP radiographs. While CT scan is considered to be superior to other methods, it measures rotation in the supine position. Atmaca et al. used the method described by Drerup to determine vertebral rotation from preoperative standing radiographs. This technique may be of use in preoperative evaluation of scoliosis and post-operative curve decompensation below the lowest instrumented vertebra [26]. Future studies comparing the different methods can be done to determine the best measure of AVR, in order to analyse the exact relationship between AVR and coronal plane deformity.

This study shows that there is an association between AVR and the coronal plane deformity. It also demonstrates that flexible curves derotate to a greater extent compared to rigid curves, when stretched.
